# The efficacy of jade moxibustion in knee osteoarthritis

**DOI:** 10.1097/MD.0000000000019845

**Published:** 2020-04-24

**Authors:** Lusheng Chen, Zouqin Huang, Ke Cheng, Fan Wu, Haiping Deng, Lin Lin, Ling Zhao, Xueyong Shen

**Affiliations:** a School of Acupuncture-Moxibustion and Tuina, Shanghai University of Traditional Chinese Medicine; b Shanghai Pudong New Area Hospital of Traditional Chinese Medicine, Pudong New Area, Shanghai, China; c Shanghai Research Center of Acupuncture and Meridian, Shanghai, China.

**Keywords:** jade moxibustion, knee osteoarthritis, pain, traditional Chinese medicine

## Abstract

**Introduction:**

This study aims to compare clinical effect between Jade moxibustion and traditional moxibustion, and to determine the clinical effect of Jade moxibustion on knee osteoarthritis (KOA).

**Methods/Design:**

This is a 2-parallel-group, randomized controlled trial. A total of 148 subjects with KOA (Kellgren–Lawrence grade II or III) will be recruited and randomized to receive Jade moxibustion treatment or a traditional moxibustion treatment in a 1:1 ratio. Jade moxibustion group: The affected knee of the subjects will be covered with jade kneepad. Traditional moxibustion group: Chosen the ST35, ST34, EX-LE4, SP10 and Ashi points at the affected knee. The subjects will receive treatment three times a week, altogether 12 times in 4 weeks. The main outcomes are WOMAC knee pain score, knee function score and SF-36 quality of life questionnaire changes at the 4th week. Secondary outcomes include WOMAC knee pain score and knee function score, overall clinical efficacy evaluation, medication, safety evaluation at the 2nd, 12th, and 24th week, and cytokines related to osteoarthritis in serum.

**Discussion:**

This randomized controlled trial used traditional moxibustion as a control group to provide rigorous evidence for the clinical efficacy and safety of Jade moxibustion in treatment of KOA.

**Trial Registration:**

ISRCTN registry, No 21174552. Registered on 28 February 2020.

## 1 Introduction

Knee osteoarthritis (KOA) is a common cause of disability in the elderly.^[1]^ In the USA, 12.1% people over 60 years of age suffered knee OA.^[2–4]^ The risk of KOA increases by age.^[5]^ The incidence of KOA is 30% in China, which women have higher risk than men.^[6,7]^ There is a correlation between knee pain and mortality in obese people. Mortality can be reduced by effective intervention, reduction of knee pain and prevention of complication.^[8]^

Nearly 23% of osteoarthritis patients with knee or hip joint suffer from neuropathic pain.^[9]^ The goals of the treatment of KOA is to reduce symptoms and ultimately slow disease progression, which may improve the quality of life and activity of subjects.^[10]^ Therefore, it is very urgent and important to find alternative and complementary methods to treat and control inflammatory pain. At present, NSAID and intraarticular corticosteroid are commonly used to relieve the pain of symptomatic osteoarthritis of the knee. NSAID has renal toxicity, serious gastrointestinal complications and cardiovascular events,^[11]^ while intraarticular corticosteroid has potential harmful effects on the knee cartilage, although both of them are helpful to relieve the pain.^[12]^

Moxibustion is a traditional Chinese medicine (TCM) method of the treatment of KOA. Unlike drug treatment, moxibustion rarely causes side effects, which can effectively relieve the pain symptoms of KOA patients,^[13–15]^ and improve the overall function.^[16–18]^ In a meta-analysis of a randomized controlled trial involving 11 moxibustion treatments for KOA, the moxibustion group had significantly improved overall pain and physical function scores compared to the traditional oral medication.^[19]^ Another study indicates that real moxibustion had effect on the osteoarthritis pain as compared to sham moxibustion.^[20]^

There have been records of using jade for treatment since ancient times in China. Inlaid in the inner side of the kneepad, the temperature of the kneepad can reach 43 to 47°C after being electrified. The results show that transient receptor potential vanilloid 1 (TRPV1) is related to the anti-inflammatory effect of the body.^[21]^ Jade moxibustion is to wrap the jade kneepad around the knee joint, which is easy to operate and can adjust the temperature. However, the therapeutic effect of Jade moxibustion on KOA has not been evaluated in clinical trials. The hypothesis is that 3 sessions per week of Jade moxibustion will have the clinical effect on KOA.

## 2 Method

### 2.1 Study design

This trial is a non-inferiority randomized controlled clinical trial, which is designed to compare the clinical efficacy of the Jade moxibustion and traditional moxibustion in treating typical inflammatory pain–KOA. The protocol has been registered on ISRCTN registry (No 21174552) and will be conducted in accordance with the Declaration of Helsinki.

**Figure 1:**
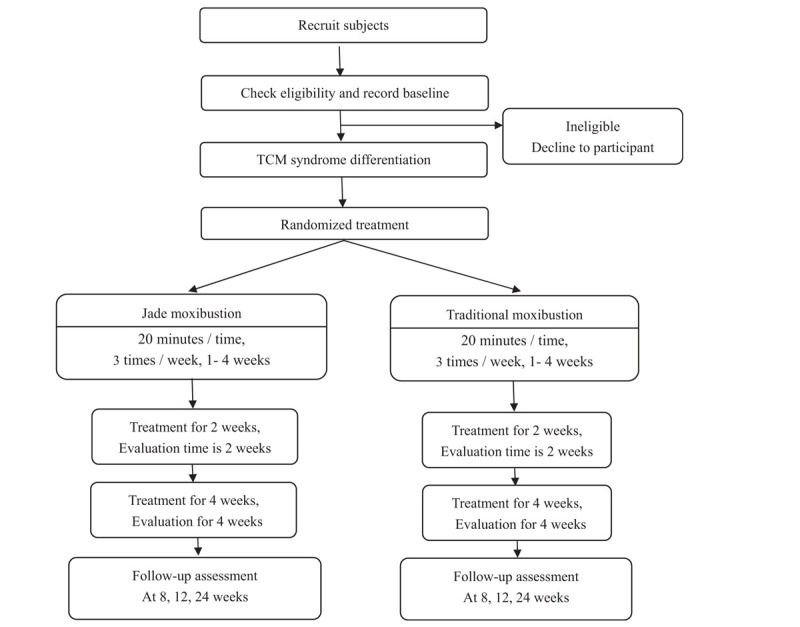
Study flow chart.

The study flow outline is shown in Figure 1

### 2.2 Study setting, recruitment, and ethics

Recruitment will be carried out in Tongren Hospital Affiliated to Shanghai Jiaotong University and Shanghai Pudong Hospital of Traditional Chinese Medicine. A total of 148 subjects with KOA will be enrolled. The study was approved by the medical ethics review committees of Tongren Hospital (No. Tongren 2019-022-02). Recruitment will be carried out through social platform, out-patient department, community propaganda and other ways. Investigator will contact people who are willing to participate in the study by telephone, conduct preliminary screening according to the inclusion and exclusion criteria, and then, if appropriate, arrange the acupuncture specialist for face-to-face baseline visit at an appropriate time, meanwhile the subjects will receive radiographic evaluation. The investigator will introduce the research protocol (i.e., the research purpose, procedures and time commitment and potential risks and benefits related to the research) in detail to potential subjects and obtain written informed consent. The subjects’ privacy information will be protected. Each subject will be given a unique random number as a direct identifier of the case report.

### 2.3 Inclusion criteria

1. Age 50 to 75 years old.
2. According to the American College of Rheumatology Diagnostic Criteria for KOA.^[22–26]^
3. Radiologic confirmation of KOA (Kallgren-Lawrence level ≥1).^[27]^
4. Moderate or greater knee pain on most days of the past month; the subject's visual analog scale baseline score for arthritic pain is 40 points and above.
5. Agreed to be randomly assigned, understand and would like to sign an informed consent.

### 2.4 Exclusion criteria

1. Knee pain caused by other diseases (such as rheumatoid arthritis, fibromyalgia syndrome, chronic fatigue syndrome and ankylosing spondylitis).
2. Treatment of steroid drugs in the past 3 months.
3. History of receiving acupuncture/moxibustion treatment within 3 months.
4. Intra-articular injection of hyaluronate over the past 6 months.
5. History of joint puncture or arthroscopy in the past year.
6. History of the knee/hip replacement surgery or planning to perform such surgery during the trial.
7. Use of other topical treatments, such as topical use of drugs.
8. Presence of any serious diseases including heart disease, lung disease, kidney disease, liver disease or malignant tumor, systemic infection or infectious disease and mental illness.
9. Participation in another clinical study in the past 1 month.

### 2.5 Withdrawal criteria

Subjects who drop or discontinue prior to completion of the treatment period should conduct a questionnaire survey and those who have serious adverse reactions will be reported to the ethics committee.

1. Those who have not completed the treatment and have withdrawn before the end of treatment.
2. Any serious adverse reactions.
3. Major problems in the protocol design or trial process (such as those who are seriously infected due to blistering).

### 2.6 Randomization

A total of 148 subjects with KOA who are eligible per protocol will be randomly assigned to group A - Jade moxibustion group or group B - traditional moxibustion group in a 1:1 ratio. The subject will first receive a brief phone consultation, after that, the subject will be allocated to the site. After the investigator and the subject signed the informed consent, the investigator will perform a simple rheumatic examination. The investigator will make a diagnosis based on the knee x-ray film brought by the subject or the knee X-ray performed at site. Only the subject who meet the inclusion/exclusion criteria will be randomized by central network randomization program. Then investigator will make baseline assessment and arrange treatment.

### 2.7 Stratification

The subjects will be stratified according to the number of lesions of the KOA (i.e., dual-joint and mono-articular).

### 2.8 Interventions

The treatment plan of the trial is based on the traditional moxibustion in the clinical treatment of KOA, combined with the characteristics of Jade moxibustion instrument itself, considering the standardized requirements of the trial design.

According to the literature review, the following 5 acupoints are selected for each treatment.ST35, ST34, EX-LE4, SP10, and Ashi point (i.e., tender point around the knee joint) are all located in the knee region, and they are common acupoints in the global clinical research of treating KOA.^[28]^ In anatomy, Acupuncture point ST35 and EX-LE4 are located close to the thinnest part of the knee joint, so the thermal effect of moxibustion and Jade moxibustion can directly reach the articular cavity. According to the biomechanical study, most of the clinical diagnosis and treatment guidelines of KOA pay special attention to the muscle strength training of quadriceps femoris.^[29–32]^ The muscle strength of extensor and flexor groups of knee joint is closely related to the symptoms of KOA.^[33]^ SP10 and ST34 are near the area of vastus medialis obliquus and lateral great respectively, so they may directly improve the state of quadriceps femoris. Ashi point is the most painful point according to the actual situation of the subjects.

Subjects are allowed to take concomitant medication, but we will encourage them not to change their medications during the trial. At the same time, other therapies are not allowed, such as acupuncture, sodium hyaluronate injection.

The treatment process will be supervised throughout by the staff of the research team. The two groups of subjects will be treated separately by the rigorously trained treatment operator.

Jade moxibustion Group

The Jade Moxibustion group will use the HX001 temperature-controlled energy knee pads produced by Jiaxing Fuqiduo Thermal Bed Co., Ltd. Jade kneecap will be electrified and preheated for 3 minutes until the temperature rises to 46°C, press the jade in the kneecap against the knee which can be fixed on the knee of the subject. If the patients felt burning at the skin, the temperature will be adjusted.

Traditional moxibustion group

The traditional moxibustion group will use stick-on moxa cone produced by Nanyang Hanyi Moxibustion Co., Ltd. The stick-on moxa cone will be ignited after being attached to the acupoints. When the patients felt burning at the skin, the moxa cone will be removed and a new one would be attached and ignited again. A small pillow will be placed in the popliteal fossa and keep the knee slightly bent and stick moxa cone on the acupoints of the subjects.

Treatment of 2 groups will last for 20 minutes, 3 times a week and altogether 12 times in 4 weeks (every Tuesday, Thursday, and Saturday). If the subject is unable to receive treatment on time, he or she will be required to complete the treatment within that week. If any adverse event occurs, treatment will be suspended, and the investigator can decide whether to terminate the treatment.

## 3 Outcomes

If the subject had a single knee joint pain, the outcome assessment will be evaluated for that knee. If both knees were affected and only one of them met the inclusion criteria, only the eligible knee will be evaluated. If both knees met the inclusion criteria, select the knee with more pain for evaluation.

### 3.1 Primary outcome indicators

The primary outcome measure is the change in pain relative to baseline at the Western Ontario and McMaster Universities Osteoarthritis Index (WOMAC) in 4 weeks. The most painful knee joint for the subject will be recorded at the baseline, which will be the joint we measured throughout the trial. We will also measure WOMAC pain at week 2, 8, 12, and 24.

### 3.2 Secondary outcome indicators

The most important secondary outcome measurement is the improvement of WOMAC function in week 4. We will also assess the total score, function score and stiffness score of WOMAC at baseline, weeks 2, 8, 12, and 24. Visual analog scale pain score will be used to evaluate the arthritis pain of subjects at baseline, weeks 2,4, 8, 12, 24. SF-36 will be used to measure the quality of life of the subjects in the 4th, 12th, and 24th week. In addition, we will evaluate the efficacy of a 5-point scale in the fourth week (1 = very poor, 2 = poor, 3 = general, 4 = OK, 5 = very good.).

Any adverse event, whether related to treatment, requires the subject and investigator to report on each treatment or visit. Side effects include redness and blisters by Jade moxibustion and traditional moxibustion. Serious adverse events will be reported to the Ethics Committee. During the follow-up period (week 5 to week 24), we will call the subject weekly to find out about the adverse reactions and side effects that may occur during the week.

After 4 weeks of treatment, investigator will ask the subjects about the safety of the treatment: safe, fewer safe, have safety problem, and have serious safety problem. After each treatment we will ask the subject's feeling: “What do you feel during the treatment? 1. Heat; 2. Cold; 3. Pain; 4. no feeling; 5. Other, please describe”

The subjects will be allowed to take the analgesic or non-steroidal anti-inflammatory drugs used before the test, we will ask subjects to record the daily dose during the entire clinical trial, and then analyze the changes in the dose.

We will measure the outcome of the baseline, week 2, 4, 8, and 12 at the hospital. For the outcome of Week 24, we will mail the questionnaire to the subjects and ask them to post them back after filling them out.

### 3.3 Exploratory outcome indicators

Exploratory outcome is the levels of IL-1β, COX-2, COMP, tumor necrosis factor IL-1α, IL-6, IL-17, IL-8, IL-4, IL-10, and IL-13 in serum. Interleukin-1b is a key virulence factor for osteoarthritis.^[34]^ IL-6, IL-8 and tumor necrosis factor alpha (TNF-A) play a role in pain during exercise, whereas TNF-A plays a role in pain at rest. The levels of Serum IL-16 were significantly associated with the WOMAC scores and their subfractions of KOA procedures and time commitments.^[35]^ The 5 ml fasting venous blood will be obtained before treatment and 4 weeks after treatment. After standing, the serum will be centrifuged, and the tubes will be placed in a refrigerator at −80°C for testing. The changes in the above markers in serum will be detected by enzyme-linked immunosorbent assay (ELISA) and Human Cytokine Array Kit. In order to reduce the influence of the circadian rhythm, each blood draw will take place between 10 and 11:30 in the morning. We will use the human cytokine array kit from R&D Systems to analyze osteoarthritis-associated cytokines in subjects’ serum. In addition to detecting the IL-1β, TNF-α, IL-6, IL-17, IL-8, IL-4, IL-10, and IL-13 cytokines, the kit can also detect additional 28 cytokines, so we can also explore whether other factors are involved in the pathogenesis of osteoarthritis.

### 3.4 Data collection and management

In order to ensure that the subjects receive a complete follow-up, all our measures are free of charge. The case report form will be completed in paper form and then entered into the spreadsheet. The original case report form and other forms (including informed consent) will be kept by the school of acupuncture and massage, Shanghai University of TCM. The required data will be collected according to the following case data collection timetable (Table 1). The Research Ethical Committee of Tongren Hospital Affiliated to Shanghai Jiaotong University will audit the trial conduct every 6 months independently of investigators and the sponsor, and will decide on any premature closure of the study.

**Table 1:**
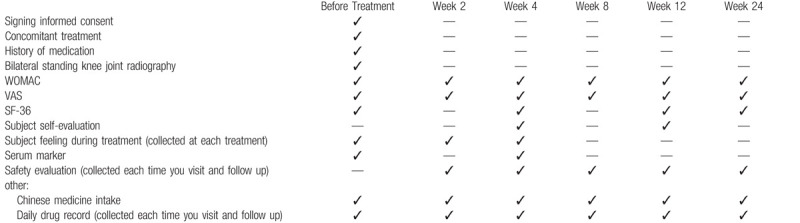
Case data collection timetable.

### 3.5 Sample size

There is study indicates that at least a 36% increase in WOMAC score is considered effective.^[36]^ In the previous study of the research group, the average effective rate of the Jade moxibustion group was 55.56%, compared with 46.51% in the traditional moxibustion group. Based on the results of our pilot study and previous reports, we used non-inferiority test to calculate the sample size (α = 67 per group) with α = 0.025, 1−β = 80%, and a cutoff of 0.5. In other words, we expect a sample size of 62 subjects per group to be sufficient to detect statistically significant differences between the 2 groups. There were no cases of shedding in the 2 treatment groups in the previous trial. In order to prevent the occurrence of shedding resulting in insufficient sample size (preset 10% shedding rate), we will recruit 74 subjects for each group, and a total of 148 subjects need to be recruited.

### 3.6 Statistical analysis

Intention-to-treat analysis is the principle of this study, which includes all subjects in the designated treatment group, as well as subjects who have completed at least one course of treatment. The significant difference level is set to 0.05. The baseline characteristics of subjects will be summarized in both groups. For continuous results, the normal distribution of the data is labeled as the mean (standard deviation). For the main outcome, the remission rate of the fourth week will be calculated and χ^2^ test will be used to compare the Jade moxibustion group with the traditional moxibustion group. For secondary outcomes, unpaired scores including WOMAC pain, functional and stiffness scores, SF-36, expectation and reliability of treatment, and overall treatment outcome will be compared at all follow-up time points in both groups. T-test or Wilcoxon rank sum test will be used to compare continuous variables. We will use SPSS version 22 (IBM SPSS Statistics, NY) for statistical analysis.

### 3.7 Ethics and publication

The study has been approved by the medical ethics review committees of Shanghai Tongren Hospital (No. Tongren 2019-022-02). The study has also been registered on ISRCTN registry (No. 15030019). Subject will be enrolled after receiving the introduction of the study and signing the informed consent form. The results of the preliminary study will be published in professional journal.

## 4 Discussion

The purpose of this study is to explore an effective moxibustion replacement therapy, Jade moxibustion for KOA. The trial will evaluate its non-inferior efficacy compared with traditional moxibustion. Subjects will receive 3 times treatment a week. For Chinese medical external treatment, the frequency of 3 times a week is the most common treatment for KOA. One limitation of this trial is that due to different treatment instrument, the operators will know about the group allocation, which may lead to subjects’ bias. Therefore, study may use the expectation scale to measure the attitude of the subject to the treatment. The trail is aimed to provide reliable evidence for Jade moxibustion in the treatment of KOA, and to make up for the lack of clinical efficacy research in this field. Also, to provide reliable data for our future multicenter randomized clinical trial. Traditional moxibustion therapy produces heavy smoke with unpleasant smell. The smoke of moxibustion is considered as a biological hazard to health,^[37]^ which is therefore prohibited from use in many clinics and hospitals. Jade moxibustion is a new therapy based on the theory of TCM and is easy to operate with no smoke, which can be further explored for treatment amount and duration.

## Author contributions

Xueyong Shen and Ling Zhao designed the study as principal investigators.

Lusheng Chen, Ke Cheng, Fan Wu, Haiping Deng, Lin Lin and Ling Zhao participated in the design of the study and drafted the manuscript.

Lusheng Chen, Zouqin Huang and Lin Lin are responsible for the recruitment and treatment of patients.

All authors read and approved the final manuscript.

Lusheng Chen orcid: 0000-0001-9618-3080.
